# Effects of temperature and soil fauna on the reduction and leaching of deoxynivalenol and zearalenone from *Fusarium graminearum*-infected maize stubbles

**DOI:** 10.1007/s12550-021-00434-y

**Published:** 2021-06-25

**Authors:** Friederike Meyer-Wolfarth, Elisabeth Oldenburg, Torsten Meiners, Katherine Muñoz, Stefan Schrader

**Affiliations:** 1 Julius Kühn-Institut (JKI), Federal Research Centre for Cultivated Plants, Institute for Plant Protection in Field Crops and Grassland, Messeweg 11/12, 38104 Braunschweig, Germany; 2 Johann Heinrich von Thünen Institute (TI) - Federal Research Institute for Rural Areas, Forestry and Fisheries, Institute of Biodiversity , Bundesallee 65, 38116 Braunschweig, Germany; 3 Julius Kühn-Institut (JKI), Federal Research Centre for Cultivated Plants, Institute for Ecological Chemistry, Plant Analysis and Stored Product Protection, Königin-Luise-Straße 19, 14195 Berlin, Germany; 4 University Koblenz-Landau, Institute for Environmental Sciences, Fortstr. 7, 76829 Landau, Germany

**Keywords:** Mycotoxins, Earthworms, Collembolans, Organic residues, Climate change

## Abstract

A microcosm study was conducted at two different temperatures under laboratory conditions to investigate the regulatory capacity and the interactive performance of two soil fauna species (*Aporrectodea caliginosa*, earthworms, and *Proisotoma minuta*, collembolans) on the reduction of *Fusarium* toxins in contaminated maize stubbles. Single and mixed species treatments were exposed to artificially infected maize stubbles highly contaminated with the mycotoxins deoxynivalenol (DON) (10,462 µg kg^−1^) and zearalenone (ZEN) (2,780 µg kg^−1^) at 17 °C and 25 °C for time periods of 3 and 6 weeks. Immediately after the respective end of incubation, the microcosms were heavily watered to determine the leaching potential of DON and ZEN from contaminated maize stubbles. Maize residues, soil, and eluted water (percolate) samples were analysed for mycotoxin content using liquid chromatography coupled to mass spectrometry. The biomass of introduced earthworms and number of collembolans were monitored to get information about their adaptability to the experimental conditions. While the decline of ZEN was temperature-dependent, but not influenced by faunal activities, a reduction of DON due to faunal impact was observed by trend. In the leaching experiment, 67–82% of the DON content in the residual maize stubbles leached from the plant material by irrigation and was detected in the soil (1.9–3.4 µg kg^−1^) and in the percolate (12–295 µg L^−1^). In the case of ZEN, 27–50% of the mycotoxin leached from the residual maize stubbles due to watering but was only occasionally detected in traces in the soil and not found in the percolate. The results clearly reveal a leaching potential of both DON and ZEN, respectively, but a mobilisation with water was only observed for DON. Temperature confirmed to be a key factor, affecting the fate of the mycotoxins in the soil by driving the interaction between different soil fauna members as well as functional and trophic levels within the soil food web.

**Supplementary information:**

The online version contains supplementary material available at 10.1007/s12550-021-00434-y.

## Introduction

Current and future agricultural plant production is facing huge challenges due to progressive change of the global climate. Climatic and weather conditions are not only important factors affecting plant growth but also influence the structure of soil inhabiting faunal and microbial communities (Guerra et al. 2021), as well as the life cycle and population development of plant pathogenic and toxigenic fungi, such as *Fusarium* spp. (Medina et al. 2017). Due to elevated atmospheric temperature and altered precipitation conditions, cultivated crops are increasingly exposed to abiotic stresses, which directly influence the plant’s defense mechanisms against fungal infections, e.g. by *Fusarium* spp., and insect pest infestation (Vaughan et al. 2018). In addition, increased pest insect infestation can lead to a rise in number of entry points for pathogens and favor, for example, *Fusarium* spp. infections and mycotoxin occurrence. Thus, a rise of yield losses due to increased occurrence of plant diseases and higher mycotoxin levels in crop products and crop residues can be expected in the future (Vaughan et al. 2016). In addition, mycotoxin contamination of crop products may induce toxic effects endangering the health of animals and humans (Bennett and Klich 2003; Rotter et al. 1996). Some authors even suggest that mycotoxins pose one of the most important food safety hazards affected by climate change (Van der Fels-Klerx et al. 2016). Despite of this threat, little is known about the occurrence and the fate of mycotoxins as environmental contaminants in water and soil compartments. Mycotoxins can enter arable soils via mobilisation by water and leaching from host tissue of infected plants and residual plant material (Elmholt 2008) and can therefore be considered as potential environmental contaminants in both water and soil (Bucheli et al. 2008; Hartmann et al. 2008; Kolpin et al. 2014). Schenzel et al. (2012a) even demonstrated that the run-off from agricultural fields is one significant source of mycotoxins in surface water.

Stem and root rot of maize caused by *Fusarium* spp. is often resulting from stress conditions like long-lasting dryness or extreme wetness weakening the plants (Dodd 1980) and may lead to multi-contamination with A- and B-type trichothecenes and zearalenone (ZEN) (Lew et al. 1997; Schollenberger et al. 2012) in infected stem parts. Concentrations of deoxynivalenol (DON) and ZEN in maize stubbles sampled from Swiss field trials at physiological maturity of the plants in late autumn ranged from 2.6 to 15.3 mg kg^−1^ and 0.7 to 7.4 mg kg^−1^, respectively (Dorn et al. 2011). Within the time period between harvest and soil tillage maize stubbles left in the field may be further contaminated with *Fusarium* toxins due to eased fungal access to the plant residues. According to the European Environment Agency (EEA 2012), observed and projected impacts from climate change for Central and Eastern Europe include an increase in warm temperature extremes and a decrease in summer precipitation, whereas for North-western Europe an increase in winter precipitation and increasing risk of river and coastal flooding is predicted. More frequent events of summer droughts or precipitation extremes are expected to promote *Fusarium* stem rot and toxin contamination of maize residues thus posing a leaching risk of *Fusarium* mycotoxins into the soil and in the drainage water and a contamination of surface water via run-off.

The fate of mycotoxins in the soil does not only depend on their chemical properties (Schenzel et al. 2012b), but largely on abiotic factors such as temperature, humidity, soil characteristics, plant growth as well as biotic influences such as the activity and composition of soil biota and their interactions (Elmholt 2008). Within the soil food web, fungal grazers among the soil fauna can be antagonistic to a *Fusarium* infection and act, therefore, as biological regulators (Turbé et al. 2010). Soil fauna interactions strongly contribute to ecosystem functioning and ecosystem services provision. They are based on competitive and facilitative effects and can be synergistic, antagonistic or just neutral. For example, it has been shown that interactions between earthworms and collembolans are complex and inconsistent (Eisenhauer 2010). Results from arable soil (Luvisol like in the present study) demonstrated an increase in collembolan numbers (hemiedaphic species *Folsomia candida*) in the presence of the endogeic earthworm species *Aporrectodea caliginosa* due to a habitat improvement by the earthworm burrow network (Wickenbrock and Heisler 1997). A laboratory study on the epi-endogeic earthworm species *Lumbricus rubellus* revealed positive effects on collembolans in forest soil through developing a pore network and providing bioavailable secretions therein (Cameron et al. 2013). The results from a study with earthworms and collembolans in forest soil mesocosms containing beech saplings suggest that soil fauna interactions mainly vary with the identity of species and community composition rather than with similarity of traits like body size or microhabitat association (Grubert et al. 2016).

It has been shown that the soil fauna is capable of suppressing the abundance of *Fusarium* spp. and of reducing the mycotoxin content substantially in arable ecosystems (Goncharov et al. 2020). For example, earthworms and collembolans are known to contribute to the biocontrol of plant pathogenic fungi such as *Fusarium* spp. and are even able to promote the reduction of mycotoxin concentrations in crop residues on the soil surface and in the soil (Meyer-Wolfarth et al. 2017a, b; van Capelle et al. 2021; Wolfarth et al. 2011a, 2013).

However, an understanding of the interactions between endogeic earthworms and hemiedaphic collembolans as well as their joined regulation capacities on *Fusarium* fungi and their mycotoxins as an interactive process is limited so far. The fate of mycotoxins in soil largely depends on many climatic factors, but particularly little is known about abiotic drivers such as temperature which affects the chemical, physical or biological interaction between mycotoxins and the soil.

Therefore, a microcosm study was conducted under laboratory conditions consisting of two consecutive experimental steps. The first step should simulate maize stubble degradation left on the soil after harvest during time courses of 3- and 6-week incubation to investigate the regulatory capacity of two soil fauna members and their interactive performance to contribute to the reduction of *Fusarium* toxins in plant residues. The endogeic earthworm species *Aporrectodea caliginosa* is one of the most widespread earthworm species in temperate regions and very common in arable soils (Lee 1985). In the present study, *A. caliginosa* is representative for secondary decomposers of the soil macrofauna with fungi as an important part of their diet (Bonkowski et al. 2000). Furthermore, *A. caliginosa* was taken as model organism for several laboratory experiments (Eisenhauer et al. 2010; Wolfarth et al. 2011b; Wurst et al. 2004). The hemiedaphic collembolan species *Proisotoma minuta* is abundant in upper soil layers and the rhizosphere of European soils (Querner et al. 2013). In the present study, *P. minuta* is representative for grazers of the soil mesofauna (Querner et al. 2013). The microcosms of the present study were exposed to two different temperatures: one set was exposed to 17 °C, which lays between the optimum temperatures of introduced earthworms (optimum about 15 °C) (Lowe and Butt 2005) and collembolans (optimum about 20 °C) (Wiles and Krogh 1998) and represents the upper limit of daytime temperature that can be actually expected in September/October for harvest of maize cultivated in Central Europe. The other set was exposed to 25 °C, which should simulate a future scenario of elevated temperature in autumn in Central Europe probably influencing the expected faunal activities and interactions. In a second step immediately after the incubation period, heavy short-term watering should simulate a sudden precipitation event to assess the leaching potential of DON and ZEN from residual maize material into the soil and the eluted water, which could pose a risk for soil health and the aquatic environment.

Our hypotheses were as follows: (1) earthworms and/or collembolans are involved in the reduction of the mycotoxin content of DON and ZEN in contaminated maize material; (2) the interaction between both faunal species results in higher DON and/or ZEN reduction rates in contaminated maize material; (3) temperature affects the faunal mycotoxins reduction efficacy and thus the contamination levels of DON and ZEN in residual maize material; (4) the mycotoxins DON and ZEN leach from contaminated maize material into the soil and are detected in the percolate (eluted water).

## Material and methods

### Soil

Topsoil was taken from a long-term experimental site at the University of Göttingen, Lower Saxony, Northern Germany, located near Göttingen (5° 29′ N, 9° 56′ E) and stored at 4 °C until further treatment. The soil type is a Haplic Luvisol derived from loess and consists of 19% clay, 68% silt and 13% sand. The pH value was 7.9. The soil was defaunated by freezing at − 20 °C for 48 h, 7 days before filling the microcosms, and thereafter thawed at room temperature for 48 h. A sterilisation of the soil was omitted for keeping the original microbial community as part of the natural food source for the introduced soil fauna (see below). Prior to the experiment, the soil was macroscopically cleared from organic material and sieved at a mesh size of 2 mm. A detailed description of soil processing is given in Wolfarth et al. (2013). The soil water content was 16% (w/w) which is equivalent to 60% of water-holding capacity. Neither DON nor ZEN was initially detected in the soil.

### Maize stubbles

Maize (*Zea mays cv.* Werena) was cultivated at an experimental site of the Julius Kühn Institute in Braunschweig (Germany). In June 2016, maize plants were inoculated by a stem injection of 0.5 mL spore suspension of *Fusarium graminearum* to induce and promote pathogen-specific fusariosis. The spore concentration of the suspension was 250,000 spores mL^−1^. For more details on inoculation, see Oldenburg and Ellner (2015). In infected stems, *F. graminearum* produced the mycotoxins DON and ZEN. The initial mycotoxin concentration of the collected and dried maize stems was 10,462 ± 80 µg kg^−1^ for DON and 2,780 ± 201 µg kg^−1^ for ZEN, respectively. Finely chopped dried maize stubbles of approximately 1.5 mm length were used for the experiment.

### Soil fauna

Adult and subadult individuals of the earthworm species *Aporrectodea caliginosa* were collected from an agricultural field as described in Wolfarth et al. (2011b) and stored at 15 °C until further processing. Ten days before starting the experiment, the earthworms were adapted to the soil conditions as recommended by Fründ et al. (2010). Before they were placed into the microcosms, the earthworms were washed with water to remove mucus and adhesive residues and soil.

The collembolan species *Proisotoma minuta* used for the experiment originated from own laboratory mass cultures. *P. minuta* was reared on a mixture of moist plaster of Paris and charcoal (9:1) and fed with brewer´s yeast and carrots twice a week. As previously described in Wolfarth et al. (2013) and Meyer-Wolfarth et al. (2017a), only young adults of the same age and size were introduced into the experimental system after starving them for 24 h.

### Experimental setup

Transparent acrylic glass cylinders (microcosms) were used as experimental units (height 12 cm, diameter 11.5 cm). The bottom openings of the cylindrically microcosms were covered with a nylon gauze (mesh size 20 µm). The microcosms were filled with 500 g dry weight (DW) soil, moistened to 60% field capacity. The resulting soil depth accounted of 6 cm should represent the top layer of soil containing microorganisms and fauna that come into close contact with plant residues on the soil surface. The soil fauna was introduced into the microcosms after filling the soil and applying 7 g of finely chopped mycotoxin-contaminated maize stubbles on the soil surface. The maize stubbles were moistened by spraying with 3 mL water, and a small portion was carefully incorporated into the uppermost cm of the soil following recommendations of Fründ et al. (2010) concerning the food supply for endogeic earthworms. During the experiment, the earthworms received no additional food. Finally, the microcosms were closed with Parafilm® M to prevent the soil fauna from escaping but enabling air exchange. There were 4 faunal treatments: earthworm treatment (4 individuals), collembolan treatment (100 individuals), mixed treatment with earthworms (4 individuals) and collembolans (100 individuals) and a control treatment without soil fauna. The experimental stocking density of earthworms slightly exceeded natural population size but followed the recommended number of individuals of *A. caliginosa* under laboratory conditions (Lowe and Butt 2005). The experiment was carried out under constant laboratory conditions over periods of 3 and 6 weeks. A total of 80 microcosms were set up for the 4 faunal treatments, 2 temperature treatments and 2 time treatments considering 5 replicates per treatment. The microcosms were randomly distributed in 2 climate chambers at 17 °C and 25 °C, respectively, in permanent darkness. Small quantities of soil and maize were retained at the beginning and stored at − 20 °C until further processing to determine the initial concentrations of the mycotoxins DON and ZEN.

### Sampling and sample processing

After 3 and 6 weeks (respective endpoints of the first experimental step), the microcosms were separated into two halves by sliding a carbon-fiber sheet/tile vertically into each microcosm. Immediately thereafter, one half of the microcosms was sampled as follows: soil samples were taken with a corer (diameter 2 cm) to extract the collembolans by using a MacFadyen high-gradient extractor (MacFadyen 1961). A detailed description of the extraction is given in Wolfarth et al. (2013). The remaining maize stubbles were sampled from the surface and soil samples were taken. Maize and soil samples were then stored at − 20 °C for analytical preparation. Finally, earthworms were removed, carefully cleaned, and weighed individually to determine their biomass.

All samples were dried in a compartment drier (Universalschrank U, Memmert, Schwabach, Germany) at 35 °C for 72 h. Maize material was ground and finely pulverised using a mixer mill (MM 400; Retsch, Haan, Germany). Samples of soil were manually homogenised with a mortar to obtain a fine powder (< 0.5 mm).

### Leaching step

The second half of the microcosms was used to obtain a leaching effect by watering their surface twice with 100 mL of tap water (200 mL in total), thus simulating a heavy short-term rainfall event of about 39 L per m^2^. Within 30 min, the water percolated through the maize layer and the soil. The percolate was collected and stored at − 20 °C in preparation for mycotoxin analysis. Thereafter, residual maize material (leached maize) and soil (leached soil) samples were taken and processed as described above before analysis.

### Mycotoxin analysis in maize, leached maize, percolate and soil

For the detection and quantification of *Fusarium* mycotoxins, DON and ZEN maize and leached maize samples of 1 g were extracted with 25 mL of a mixture of acetonitrile/water (50/50, v/v) by turbulent overhead shaking for 30 min (Gerhardt RA 20, Bonn, Germany). The extracts were filtered (Falcon© Filter Roth CA18.1) and then diluted 1:10 with 30% methanol. Ten microliters of the purified and diluted filtrate were analysed by HPLC with mass spectrometry detection (see below). After defrosting and filtering (Falcon© Filter Roth CA18.1), percolate water samples were concentrated and purified by performing reversed phase solid-phase extraction (Chromabond HR-P cartridges, 3 mL, 200 mg; Macherey–Nagel). Analytes were eluted with 5 mL of methanol, reduced to 100 ± 5 μL, and reconstituted in 1000 μL of Milli-Q water/methanol (90:10, v/v). Ten microliters of the samples were analysed by HPLC with mass spectrometry detection. A Thermo Scientific DIONEX UltiMate 3000 HPLC system was used for running the maize stubble and percolate water extracts. The column was a Phenomenex Kinetex C18 (2.6 µm, 100 mm, 3 mm i.d.). The mobile phase consisted of solvent A (methanol + 0.5% acetic acid + 5 mmol ammonium acetate) and solvent B (water + 0.5% acetic acid + 5 mmol ammonium acetate). A gradient procedure was used as follows: starting with 2% of solvent A up to 98%. The flow rate was 300 μL min^−1^ and the column temperature was set at 40 °C. The HPLC was coupled with the mass spectrometer QTRAP 5500 (AB SCIEX) that was used in electrospray ionisation mode. For quantification the use of matrix-matched calibration with internal standards was necessary (range 1–1000 µg L^−1^). The limit of detection was calculated as concentrations corresponding to a signal-to-noise ratio (S/N) of 3:1 being 10 μg kg^−1^ for DON and 4 μg kg^−1^ for ZEN, respectively. For further information on mycotoxin determination, see Oldenburg and Ellner (2015).

Mycotoxins in soil samples (5 g) were extracted with 15 mL of a methanol/water mixture (9:1, v/v) for 30 min on a horizontal shaker (Kreisschüttler 3015, GFL, Burgwedel, Germany) and subsequently for 10 min with ultrasonication (DT 514H, Bandelin electronics GmbH & Co.KG, Berlin, Germany). The mixture was centrifuged for 10 min at 2000* g* (Universal 320, Hettich Lab Technology, Tuttlingen, Germany) and an aliquot of 10 mL was subsequently evaporated to dryness under a nitrogen stream at 50 °C (Evaporatorsystem EVA-EC1-24-S, VLM Korrosions-Prüftechnik, Labortechnik & Dienstleistungen GmbH, Bielefeld, Germany). Sample extracts were reconstituted in 1 mL of solution consisting of methanol/water 1:1 v/v conditioned with 0.1% formic acid and 4 mM ammonia formate, which corresponds to the composition of the mobile phase. The reconstituted extracts were ultra-centrifuged for 5 min at 7270* g* (Micro centaur, MSE Ltd, London, UK) and 20 µL of the supernatant were injected in the LC-HRMS Orbitrap-Exactive (Thermo Fisher Scientific, Waltham, USA), in electrospray ionisation mode. Chromatographic separation was performed in a Hypersil GOLD™ C_18_ column (100 × 2.1 mm, 1.9 μm particle size, Thermo Fisher Scientific, Waltham, USA). The mobile phase consisted of methanol (solvent A) und milli-Q Water (solvent B), both conditioned with 0.1% formic acid and 4 mM ammonium formate in a stepwise gradient: 0–1 min 100% B, 2–6 min 0 to 100% A, 5 min isocratic 100% A, and 12–15 min 100% B (re-equilibration). The flow rate was set at 0.2 mL min^−1^. The mycotoxins were quantified with a matrix-matched calibration curve (range 1–300 µg L^−1^). The limit of quantification of the method was calculated based on the lowest calibration level (LCL) of the matrix matched calibration curve and corresponds to 0.75 and 0.3 µg kg^−1^ for DON and ZEN, respectively. DON and ZEN were quantified in the negative ion mode, using the mass-to-charge ratios: 341.1240 and 317.1389. ^13^C-Deoxynivalenol (m/z 356.1750) was used for the confirmation of DON in soil samples. For a detailed description of the quality parameters of the method see Mortensen et al. (2006) and Muñoz et al. (2015).

### Statistics

All statistical analysis was done in R (R Development Core Team 2018) using libraries, nlme (Pinheiro et al. 2018), desplot (Wright 2018), emmeans (Lenth 2018), lmerTest (Kuznetsova et al. 2017), and ggplot2 (Tang et al. 2016; Wickham 2016). Requirements of normality and heterogeneity of variance were tested by diagnostic plots (Residual variances, QQ plot, and Cook distance plot). For the data set of DON in maize (unleached and leached) a square root transformation was applied. Data sets of ZEN in maize (unleached), DON in soil, as well as the data for biomass and individual numbers of soil fauna were log10 transformed. Linear models (LMs) were performed for all data sets. Analysis of variance (ANOVA) was carried out to assess the effects of the factors “soil fauna”, “time” and “temperature” on the DON and ZEN concentration in maize stubbles (leached and unleached), soil and water as well as on biomass and individual numbers of soil fauna. The level of significance was chosen to be 5%. In the cases of non-significant three-way and two-way interactions, the model was simplified by performing stepwise backward elimination. This step was conducted for the data of ZEN in maize stubbles, DON in leached maize stubbles and collembolans. To assess treatment effects of the significant factors, pairwise comparisons were conducted.

## Results

### Microcosm experiment: effects of soil fauna, time and temperature

#### DON in maize stubbles

**Fig. 1:**
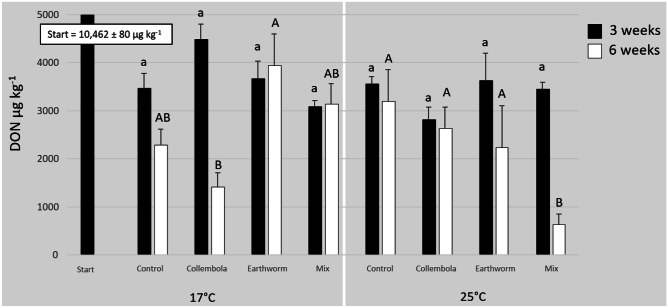
Mean (+ SE) concentrations of deoxynivalenol (DON) (µg kg^−1^) in contaminated maize stubbles on the soil surface of the microcosms in different faunal treatments: non-faunal “Control”, “Collembolans”, “Earthworm” and “Mix” at the beginning (“Start”) and after 3 and 6 weeks of inoculation separated for the different temperatures (17 °C and 25 °C) (number of replicates: *n* = 5). Different letters indicate bars to be significantly different (*P* < 0.05). Small letters refer to the bars of 3 weeks and capital letters to the bars of 6 weeks

ANOVA for the DON concentration in maize stubbles revealed significant effects for the factor “time” (Table 1). The two-way interaction of “soil fauna” × “temperature” and the three-way-interaction of “soil fauna” × “time” × “temperature” were also significant. For the factors “soil fauna” and “temperature”, the ANOVA revealed a trend towards a significant effect considering a significance level of 10%. The initial concentration of DON in maize stubbles was 10,462 ± 80 µg kg^−1^. After 3 weeks at 17 °C, the DON concentration was reduced significantly in all treatments (average decrease 65%) compared to the start, but no statistical differences could be determined between all soil fauna treatments and the control (Fig. 1). After 6 weeks at 17 °C, DON was reduced to a higher extent resulting in an average decrease of 74%. The lowest DON concentration was detected in the collembolan treatment, which differed significantly from the earthworm treatment but not from the mixed and the control treatment (Fig. 1). After 3 weeks at temperature exposition to 25 °C, a slightly higher average reduction of DON concentration (68%) in the stubbles could be detected compared to 17 °C. But again no statistical differences were found between all the treatments (Fig. 1). After 6 weeks at 25 °C, the average reduction of DON was 79%. The lowest concentration of DON in the stubbles could be measured in the mixed treatment, which significantly differed from all other soil fauna treatments and the control (Fig. 1).

**Table 1:** *F*-values, *p* values and degrees of freedom (*df*) of the multifactorial ANOVA on the effects of soil fauna, time, and temperature on the DON concentration in maize, maize leached, percolate and soil leached. *p* Values in bold are significant

	Maize			Maize leached			Percolate			Soil leached		
	*df*	*F*-value	*p* value	*df*	*F*-value	*p* value	*df*	*F*-value	*p* value	*df*	*F*-value	*p* value
Soil fauna	3	2.424	0.074	3	0.309	0.819	3	6.740	** < 0.001**	3	2.451	0.077
Time	1	31.489	** < 0.001**	1	0.402	0.528	1	181.756	** < 0.001**	1	152.319	** < 0.001**
Temperature	1	3.491	0.066	1	0.452	0.504	1	10.305	** < 0.010**	1	2.471	0.124
Fauna × time	3	1.574	0.204				3	3.908	** < 0.050**	3	2.448	0.077
Fauna × temperature	3	3.139	** < 0.050**				3	0.845	0.475			
Time × temperature	1	0.508	0.478				1	0.005	0.945			
Fauna × time × temperature	3	9.177	** < 0.001**				3	4.589	** < 0.010**

#### ZEN in maize stubbles

**Fig. 2:**
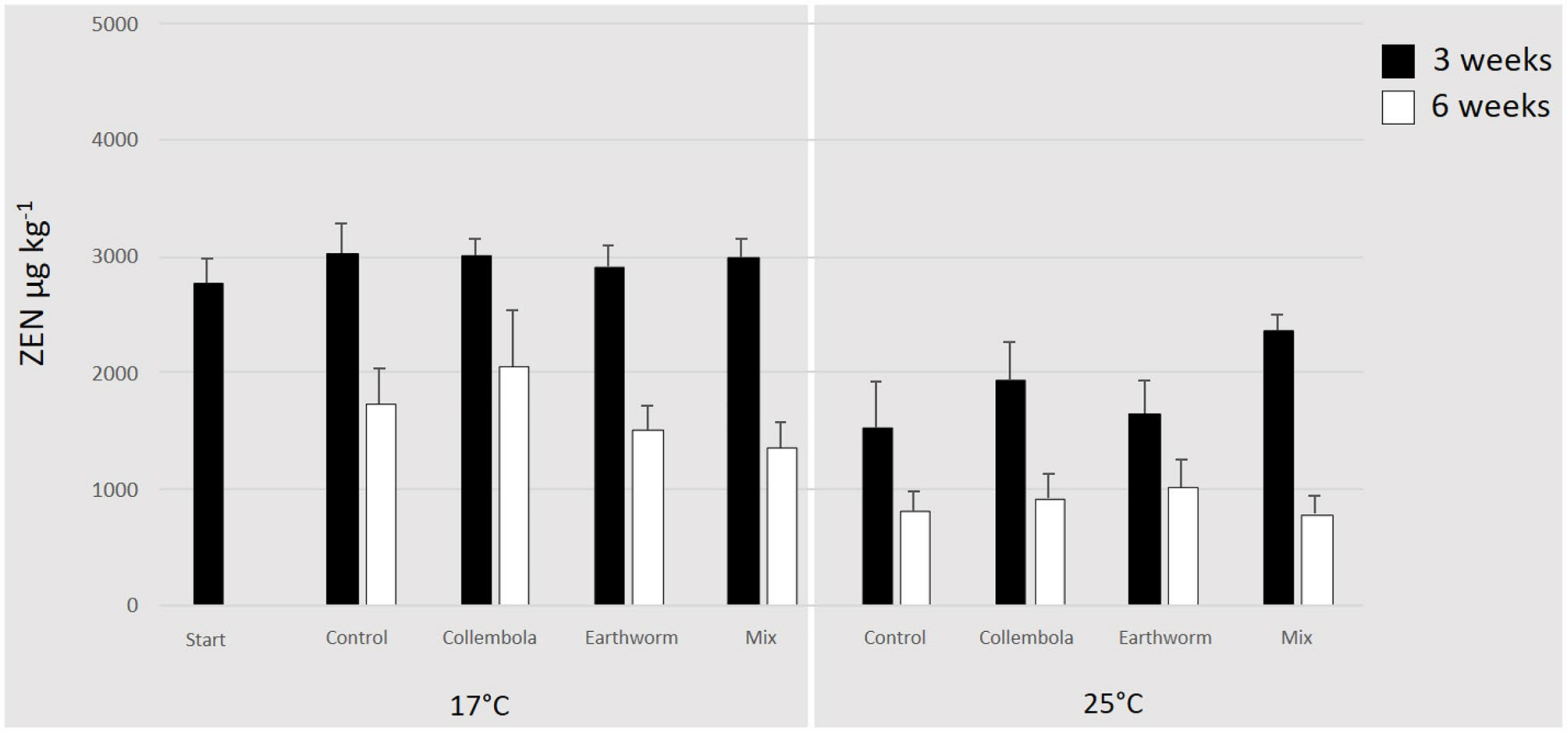
Mean (+ SE) concentrations of zearalenone (ZEN) (µg kg^−1^) in contaminated maize stubbles on the soil surface of the microcosms in different faunal treatments: non-faunal “Control”, “Collembolans”, “Earthworm” and “Mix” at the beginning (“Start”) and after 3 and 6 weeks of inoculation separated for the different temperatures (17 °C and 25 °C) (number of replicates: *n* = 5)

For the dataset of ZEN in maize stubbles ANOVA revealed significant effects for the factors “time” and “temperature” (Table 2). The starting concentration of ZEN in maize stubbles was 2,780 ± 201 µg kg^−1^. After 3 weeks at 17 °C, the ZEN concentration in the stubbles was slightly increased by 8% on average throughout all treatments compared to the start, whereas after 6 weeks at 17 °C, ZEN was clearly reduced resulting in a mean decrease of 40% (Fig. 2). At a temperature of 25 °C, ZEN concentration led to a mean significant reduction of 32% after 3 weeks. After 6 weeks at 25 °C, the ZEN concentration decreased further to about 68%. The different soil fauna treatments did not reduce ZEN significantly when compared to the control (Fig. 2).

**Table 2:** *F*-values, *p* values and degrees of freedom (*df*) of the multifactorial ANOVA on the effects of soil fauna, time, and temperature on the ZEN concentration in maize and maize leached. *p* Values in bold are significant

	Maize			Maize leached		
	*df*	*F*-value	*p* value	*df*	*F*-value	*p* value
Soil fauna	3	0.331	0.803	3	0.781	0.509
Time	1	42.032	** < 0.001**	1	82.353	** < 0.001**
Temperature	1	26.102	** < 0.001**	1	4.975	** < 0.050**
Fauna × time				3	2.996	** < 0.050**
Fauna × temperature				3	1.230	0.310
Time × temperature				1	0.020	0.888
Fauna × time × temperature				3	2.494	0.068

#### DON and ZEN in soil

Neither DON nor ZEN could be detected in the soil of any of the microcosms after 3 or 6 weeks.

### Biomass and individual density of soil fauna

#### Earthworms

The initial biomass of earthworms per microcosm varied between 0.96 and 1.25 g (fresh weight). The statistical analysis revealed significant effects for all factors: “soil fauna” (*F*-value = 26.3; *p* value < 0.001), “time” (*F*-value = 169.9; *p* value < 0.001), and “temperature” (*F*-value = 5.9; *p* value = 0.021). Earthworm biomass decreased throughout all microcosms. This decrease was generally higher after 6 weeks compared to 3 weeks of incubation at both temperature treatments (Table 3). This corresponded to the different recapture rates recorded in dependence of incubation time, which was 93% at 17 °C, but 88% at 25 °C. At 17 °C a slightly higher biomass reduction was observed in the mixed species compared to the single species treatments. An opposite pattern was found at 25 °C where higher biomass losses were recorded in the single species than in the mixed species treatments. The highest earthworm biomass reduction was 33% in the single species treatment after 6 weeks at 25 °C.

**Table 3:** Relative biomass decrease (%) of earthworms per microcosm and individual number of collembolans after 3 and 6 weeks in single- and mixed-species treatments

	17 °C		25 °C	
Treatment	Biomass of earthworms decrease (%)	Number of collembolans (absolute)	Biomass of earthworms decrease (%)	Number of collembolans (absolute)
**3 weeks**				
Control	-	-	-	-
Collembolans	-	393	-	801
Earthworms	20	-	25	-
Mix	28	358	24	1531
**6 weeks**				
Control	-	-	-	-
Collembolans	-	6738	-	6652
Earthworms	27	-	33	-
Mix	32	2089	29	2289

#### Collembolans

The statistical analysis revealed significant effects for all factors: “soil fauna” (*F*-value = 5.2; *p* value = 0.030), “time” (*F*-value = 102.8; *p* value < 0.001) and “temperature” (*F*-value = 9.9; *p* value = 0.003). The two-way interaction of “soil fauna” × “time” (*F*-value = 15.3; *p* value < 0.001) as well as the interaction of “time” × “temperature” (*F*-value = 8.3; *p* value = 0.007) was also significant. The initial number of collembolans was 100 individuals per microcosm. An increase of individual numbers of collembolans could be recorded in all microcosms (Table 3). After 3 weeks, the increase seemed to be strongly influenced by temperature since the individual numbers were twice and three times higher, respectively, in the 25 °C treatments compared to the 17 °C treatments. After 6 weeks, there was a 67-fold increase of individual numbers in the single species treatments regardless of temperature. In the mixed species treatments, a 20-fold increase of individual numbers of collembolans was measured, which seemed to be independent from temperature as well. Consequently, the development of collembolans seemed to be affected by faunal interaction effects after a longer experimental timespan of 6 weeks.

### Leaching step

#### DON in leached maize stubbles

**Fig. 3:**
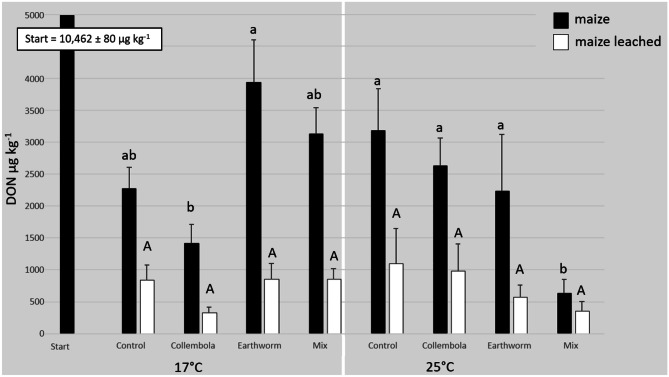
Mean (+ SE) concentrations of deoxynivalenol (DON) (µg kg^−1^) in contaminated maize stubbles on the soil surface of the microcosms before (maize) and after (maize leached) the leaching step in different faunal treatments: non-faunal “Control”, “Collembolans”, “Earthworm” and “Mix” at the beginning (“Start”) and at the 6-week endpoint of the experiment separated for the different temperatures (17 °C and 25 °C) (number of replicates: *n* = 5). Different letters indicate bars to be significantly different (*P* < 0.05). Small letters refer to the bars of “maize” and capital letters to the bars of “maize leached”

The factors “soil fauna”, “time” and “temperature” had no significant effects on the concentration of DON in maize stubbles after the leaching event, as calculated by the ANOVA (Table 1). The concentration of DON in maize stubbles after leaching at the 3-week endpoint of the experiment at 17 °C varied between 680 (± 74) and 904 (± 201) µg kg^−1^. In the 25 °C treatments DON concentrations of the leached maize stubbles varied between 528 (± 58) and 684 (± 127) µg kg^−1^. Figure 3 shows the concentrations of DON in the leached maize stubbles after 6 weeks at 17 °C and 25 °C in comparison to the non-leached maize (microcosm experiment). The mean loss of DON from maize stubbles after the leaching event, compared to the non-leached maize, was 72% at 17 °C and 68% at 25 °C.

#### DON in percolate and soil

Concerning the leaching water (percolate), the ANOVA revealed significant effects for all three factors “soil fauna”, “time” and “temperature” as well as for the two-way interaction of “soil fauna” × “time” and the three-way interaction on the DON concentration. After 3 weeks at 17 °C, a DON concentration of 180 (± 22) µg L^−1^ (non-faunal control), 155 (± 8) µg L^−1^ (collembolans), 248 (± 15) µg L^−1^ (earthworms) and 208 (± 15) µg L^−1^ (mix) has been determined in the percolate. In the percolate of the 25 °C treatments, DON concentrations of 176 (± 10) µg L^−1^ (non-faunal control), 110 (± 9) µg L^−1^ (collembolans), 171 (± 32) µg L^−1^ (earthworms) and 216 (± 14) µg L^−1^ (mix) were measured. The statistical analysis revealed significant effects for the factor “time” on the DON concentration in the soil after the leaching event (Table 1). The concentration of DON in the soil after leaching after 3 weeks at 17 °C varied between 1.9 and 2.4 (± 0.2) µg kg^−1^. In the 25 °C treatments, DON concentrations detected in the soil varied between 1.5 (± 0.4) and 3.4 (± 0.7) µg kg^−1^.

Associating the initial load of DON introduced with the maize stubbles into the microcosms, a mass balance of DON was calculated from the detected concentrations in residual maize, percolate and soil after leaching, which is shown in Table 4. When microcosms were leached after 3 weeks of incubation at 17 °C, DON was found at high levels in the percolate, but at very small levels in the soil. In total, more DON was washed out than initially loaded (control 106%, earthworm 144%, mix 121%), except for the collembolan treatment showing slightly less DON recovery (95%) in comparison with the initial load. A similar distribution pattern was resulting from leaching after 3 weeks of incubation at 25 °C, showing high amounts of DON in the percolate leading to exceeding percentages of DON recovered in control (104%), earthworms (101%) and mix (125%) compared with the initial load. By contrast at 17 °C, the DON level in the percolate of the collembolan treatment was considerably lower at 25 °C leading to a markedly less total DON recovery (67%).

**Table 4:** Mass balance calculation of the absolute DON concentration (µg per half microcosm) of the different treatments (control, collembolans, earthworms and mix)

Treatment	17 °C				25 °C			
	3 weeks		6 weeks		3 weeks		6 weeks	
	DON (µg)	DON (% of initial load)	DON (µg)	DON (% of initial load)	DON (µg)	DON (% of initial load)	DON (µg)	DON (% of initial load)
**Control**								
Maize leached (Ml)	2.38	6	2.96	8	2.17	6	3.85	11
Soil (S)	0.59	2	2.34	7	0.85	2	2.3	6
Percolate (P)	35.99	98	15.45	42	35.15	96	5.58	15
∑ Ml + S + P	38.96	106	20.75	57	38.17	104	11.73	32
**Collembolans**								
Maize leached (Ml)	3.16	8	1.18	3	2.18	6	3.44	9
Soil (S)	0.56	2	4.25	12	0.37	1	2.63	7
Percolate (P)	31.03	85	9.08	25	22.04	60	13.76	38
∑ Ml + S + P	34.75	95	14.51	40	24.59	67	19.83	54
**Earthworms**								
Maize leached (Ml)	2.66	7	3.02	8	2.39	7	2.02	6
Soil (S)	0.53	2	2.9	8	0.53	1	1.39	3
Percolate (P)	49.65	135	15.67	43	34.17	93	12.34	34
∑ Ml + S + P	52.84	144	21.59	59	37.09	101	15.75	43
**Mix**								
Maize leached (Ml)	2.38	6	3.02	8	1.84	5	1.24	3
Soil (S)	0.49	1	3.45	10	0.82	2	2.81	8
Percolate (P)	41.59	114	18.05	49	43.12	118	2.49	7
∑ Ml + S + P	44.46	121	24.52	67	45.78	125	6.54	18

When the leaching step followed the 6 weeks incubation time, much less DON was washed out at both temperatures in the percolate and similar low amounts remained both in soil and leached maize stubbles, respectively, compared to the 3-week incubation time. In consequence, the total DON content, which was recovered from the percolate, soil and maize stubbles after leaching, was considerably less in all treatments after 6 weeks incubation time than initially loaded. DON recovery rates were higher at 17 °C (control 57%, collembolans 40%, earthworms 59%, mix 67%) compared to 25 °C (control 32%, collembolans 54%, earthworms 43%, mix 18%) except for the collembolan treatment.

#### ZEN in leached maize stubbles

**Fig. 4:**
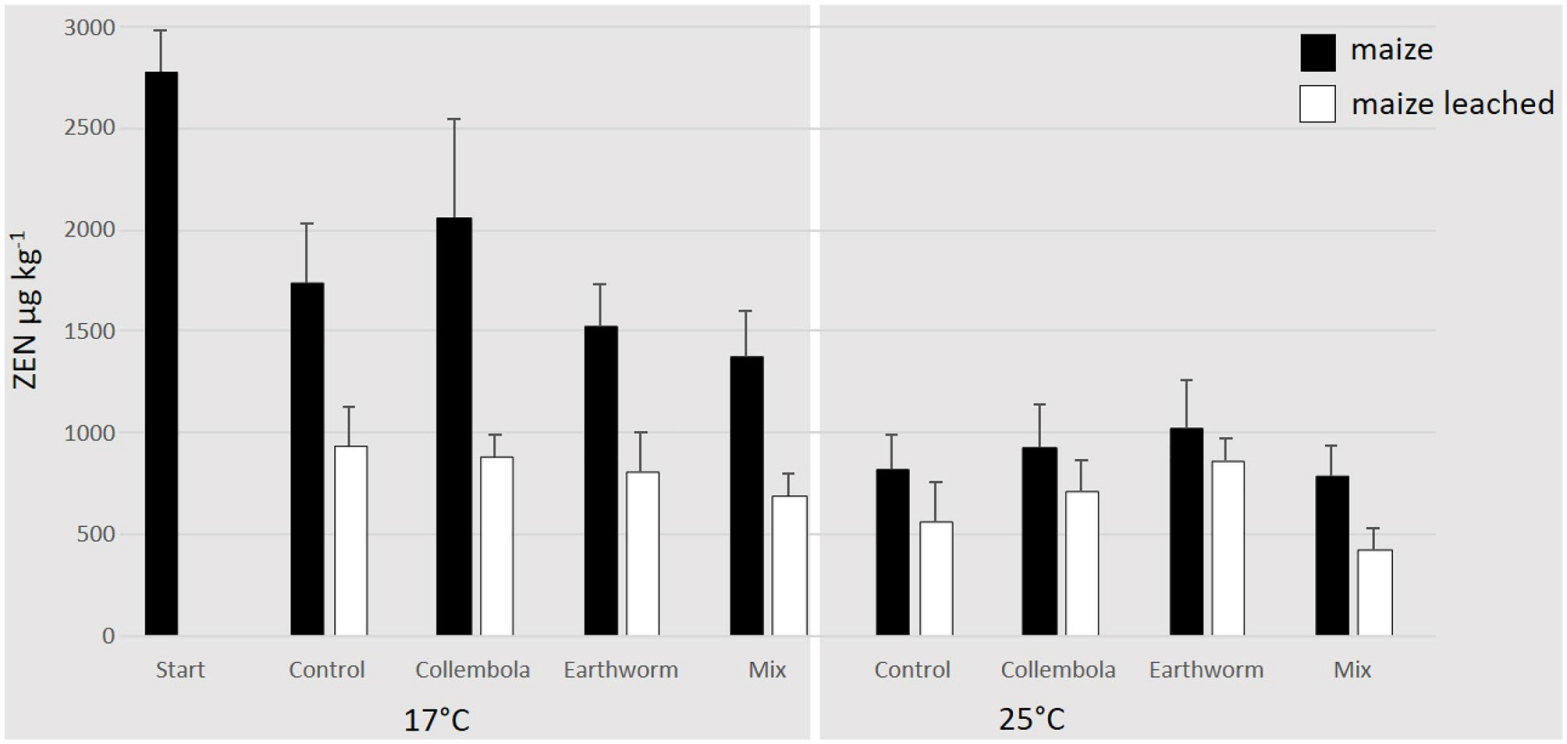
Mean (+ SE) concentrations of zearalenone (ZEN) (µg kg^−1^) in contaminated maize stubbles on the soil surface of the microcosms before (maize) and after (maize leached) the leaching step in different faunal treatments: non-faunal “Control”, “Collembolans”, “Earthworm” and “Mix” at the beginning (“Start”) and at the 6-week endpoint of the experiment separated for the different temperatures (17 °C and 25 °C) (number of replicates: *n* = 5)

For the dataset of ZEN in leached maize stubbles, ANOVA revealed significant effects for the factors “time” and “temperature” as well as for the two-way-interaction of “soil fauna” × “time” (Table 2). The concentration of ZEN in maize stubbles after leaching at the 3-week endpoint of the experiment at 17 °C varied between 1,141 (± 268) and 1,886 (± 230) µg kg^−1^. In the 25 °C treatments, the ZEN concentrations of the leached maize stubbles varied between 1155 (± 86) and 1617 (± 106) µg kg^−1^. Figure 4 shows the ZEN concentration of the leached maize stubbles compared to the non-leached maize after 6 weeks at 17 °C and 25 °C. Compared to the non-leached maize, the mean loss of ZEN in maize stubbles after the leaching event was 50% at 17 °C and 29% at 25 °C.

#### ZEN in percolate and soil

ZEN could not be detected in the soil and in the percolate from any microcosm.

## Discussion

### Microcosm experiment

The presence of the earthworm and collembolan species in the single faunal treatments did not facilitate a reduction of DON concentration in the maize stubbles observed both after 3 and 6 weeks. It was the mixed faunal treatment with interacting earthworms and collembolans (at 25 °C after 6 weeks), which reduced the DON concentration significantly compared to the non-faunal control treatment. There is an increasing consent that soil processes and the provision of soil services such as regulation of toxic contaminants is affected by soil fauna interactions (Wall et al. 2012). The endogeic species *A. caliginosa* moves to hot spots of decomposing organic material to feed on this material. The earthworms also feed on the soil microbial community, which develops there, and on egestions of other soil fauna like collembolans (here: *P. minuta)*. Since organic amendments at the topsoil are known to stimulate fungal growth (Tu et al. 2006), the decomposing chopped maize stubbles in the experiment possibly formed such hotspots, which support the mobilisation of the soil fauna from below to the topsoil. In addition, the higher environmental temperature (25 °C) accelerates the microbial activity and thus the decomposition of organic material, which probably made it more attractive for the earthworms and the collembolans in the present study.

The experimental time span had an effect on the DON degradation, as the DON concentration in maize residues was lower at 6 weeks compared to 3 weeks of incubation. This in turn can also be explained by the decomposition of organic material, which occurs with a time delay and may generate an optimum nutrient status in the soil by promoting microbial growth and activity. Temperature affected the DON concentration only slightly in combination with either soil fauna or soil fauna and time, showing the lowest DON concentration in residual maize of the mixed species treatment after 6 weeks of incubation at 25 °C.

In contrast to DON, the concentration of ZEN in residual maize was significantly influenced by the temperature as a single factor, as ZEN reduction was constantly higher at 25 °C compared to 17 °C. In accordance with the reduction of DON, time significantly influenced ZEN reduction, since lower concentrations were determined after 6 weeks compared to 3 weeks. No single or interactive faunal effect could be observed concerning ZEN degradation in any microcosm.

Therefore, our first hypotheses that earthworms and/or collembolans are involved in the reduction of DON and ZEN in contaminated maize material as individual actors cannot be confirmed in this study. The second hypothesis expecting earthworm-collembolan-interactions promoting DON and ZEN reduction in contaminated maize material is only confirmed for DON in the specific case of long-term faunal exposure to 25 °C. In contrast to this finding, a former microcosm study reported a more consistent interactive contribution of different soil fauna species (collembolans and nematodes) reducing DON in contaminated wheat straw significantly more than single species treatments (Wolfarth et al. 2013). In a mesocosm study under field conditions, an earthworm (*Lumbricus terrestris*)-collembolan (*Folsomia candida*)-nematode (*Aphelechoides saprophilus*)-combination had no promoting effect on DON reduction in wheat straw (Wolfarth et al. 2016); here, the burrowing earthworm species *L. terrestris* was confirmed to be the driver in the DON degradation process. This was also demonstrated by Abid et al. (2021) showing an accelerated disappearance of DON from contaminated wheat straw in the presence of *L. terrestris*, especially when the straw was incorporated into the soil. In the present study, the earthworm biomass decreased with time of exposure and increase of temperature thus being in line with the preference of *A. caliginosa* for cooler temperatures. The observed biomass loss of *A. caliginosa* during the experiment is still acceptable since it is in line with findings of previous studies of Oldenburg et al. (2008), Schrader et al. (2009) and Wolfarth et al. (2011a, b) where a decline of earthworm biomass was detected. According to Fründ et al. (2010), an earthworm biomass loss of approximately 30% (or less) indicates still appropriate conditions especially for long-term experiments (longer than 2 weeks). In contrast to the biomass of *A. caliginosa*, the number of collembolans increased constantly compared to the start showing a better adaptive capacity to the experimental conditions. In a previous study of Wolfarth et al. (2013), collembolans (*Folsomia candida*) were exposed to mycotoxin-contaminated and non-contaminated wheat straw in a minicontainer approach. Here the population of collembolans exposed to non-contaminated wheat straw grew faster compared to that of collembolans, which fed on contaminated straw. Szabo et al. (2019) reported mortality and high impairment of reproduction of *Folsomia candida* due to feed refusal caused by T-2- and DON-contaminated maize food substrate. However, results of Wolfarth et al. (2015) could not confirm a clear tendency towards a lower individual number of collembolans fed on mycotoxin-contaminated wheat straw compared to non-contaminated treatments. In the mixed species treatment, the individual density of collembolans was considerably lower after 6 weeks at both temperatures than in the single species treatments. This is in line with findings of Eisenhauer (2010) showing that in particular endogeic earthworm species affect microarthropods mainly in a negative manner due to the competition for food resources. In reverse, the earthworms seemed to be positively influenced by the presence of collembolans in the 25 °C treatments, as the biomass loss was mitigated in the mixed species compared to the single species treatments.

It is supposed that DON and ZEN reduction observed in maize stubbles during microcosm incubation was associated to maize stubble decomposition initiated by microorganisms active in the non-sterilised experimental soil used in our study. A microcosms study of Liebich et al. (2007) investigating microbial degradation and humification of maize straw in a sterilised arable soil (Orthic Luvisol) revealed most efficient carbon mineralisation of about 41% within 6 weeks of incubation at 20 °C when a natural complex microbial community was added. Previous studies have shown that soil microbiota has a positive impact in the degradation or mineralisation of DON (Karlovsky 2011; Sato et al. 2012; Völkl et al. 2004) and ZEN in the soil (Mortensen et al. 2006; Tan et al. 2014). As processes in soils are reciprocal and complementary, it can be assumed that soil microbiota contributes to DON and ZEN degradation, but soil fauna, as shown in the present study, improve the potential of the soil for mycotoxin degradation or mineralisation. As already discussed above, the residual maize stubbles as organic amendments could improve the degradation processes in the interface of the plant residues and the topsoil thus contributing to a higher microbial and biological activity (Schirmel et al. 2018). The capacities of microbial consortia and bacterial as well as fungal isolates derived from soil with respect to biodegradation of DON and ZEN are well documented (Shima et al. 1997; Tan et al. 2014; Ji et al. 2016; Zhai et al. 2019; Wang et al. 2020). In a recent microcosm study, DON was shown to modify the community structures of soil bacteria, fungi and protozoa to various extents, depending on the incorporation degree of straw residues and/or the presence of earthworms (Abid et al. 2021). However, the complex interactive processes between the soil microbiota and fauna regarding the decomposition of organic matter and degradation of toxic metabolites should be further elucidated for a better understanding of their respective functional roles.

The temperature is known to have an important influence on the composition and the activity of microbial communities and interactions with other members of the soil food web. Increasing the temperature from 17 to 25 °C in our study resulted in a slightly lower DON and significantly lower ZEN level in residual maize material. In consequence, our third hypothesis that temperature affects the reduction efficacy and thus the concentration of mycotoxins, can be confirmed only for ZEN in residual maize material.

### Leaching step

The mycotoxin DON leached to an extent of 68–72% from the contaminated maize material into the percolate water, but only small amounts remained in the soil. These results confirm findings of a greenhouse experiment from Gautam and Dill-Macky (2012), who measured the leaching of DON from infected plant tissue to an extent of 36–52% after a single wetting event and detected DON and 15-acetyl-DON (15-ADON) in the run-off water. The extend of leaching in the present study was detected to be almost double than that in the study of Gautam and Dill-Macky (2012), which can be attributed to the considerably higher DON contamination of the introduced plant material. Under field conditions, Ribeiro et al. (2016) postulated leaching of *Fusarium* mycotoxins from infected crops, as DON and ZEN were detected in water samples of the Duoro River in Portugal. The authors reported higher levels of DON in the river water particularly during spring and summer as a result of the local agricultural practice in combination with proper climatic conditions.

Mass balance calculations regarding the fate of DON introduced with the contaminated maize stubbles showed that more DON than initially loaded was washed out into the percolate after 3 weeks of incubation at both temperatures. The collembolan treatment was the only exception showing a 95% recovery at 17 °C and a 67% recovery at 25 °C compared to the initial DON load. As only low amounts of DON remained in leached maize material and soil, no explanation can yet be given on which biochemical and metabolic conversions or physical influences are responsible for the observed excessive run-off of DON. In contrast, DON concentrations detected in the percolate were considerably lower after 6 weeks of incubations at both temperatures, thus leading to considerably reduced recoveries of DON compared to the initial load. After 25 °C incubation DON recoveries were smaller (mean of all treatments 37%) than after 17 °C incubation (mean of all treatments 57%). Low DON concentrations remained in leached maize material and soil as well, so about half of the initial DON load must have been metabolised or masked or immobilized in soil. Again a collembolan effect was observed assuming a contribution of this species in the overall metabolic turnover of DON, as the lowest recovery rates were resulting from the collembolan treatment at 17 °C (40%) and the mixed treatment at 25 °C (18%).

Compared to DON, the leaching potential of ZEN revealed to be much less (29–50%). Furthermore, ZEN could not be detected in the soil and the percolate. The differences between the eluting behaviour of ZEN and DON can be explained by their physicochemical properties. As Schenzel et al. (2012a, b) stated, the hydrophobicity (or the hydrophilicity) can be expressed by the soil organic matter-water distribution coefficient (Log *K*_oc_) or the octanol–water partitioning coefficient (Log *K*_ow_). Hydrophilic substances, such as DON, are mobile and expected to be least retarded in the soil passage and to elute the most. In contrast, ZEN is known to be more hydrophobic and thus to be substantially retained in the soil. ZEN may be immobilised in the soil by adsorption to soil particles as the ability of clay minerals to adsorb mycotoxins such as ZEN is well documented (Elmholt 2008). For other mycotoxins such as fumonisin B1, Williams et al. (2003) suggested that in complex soils, mycotoxins are expected to be retained in the soil matrix more likely. Furthermore, the pH value may affect the mobilisation/immobilisation of mycotoxins in the soil as binding occurs via ionic interactions with soil particles. Acidic conditions (lower pH values) may promote the mobilisation. The pH value of the soil in the present experiment was 7.9, which increases the probability of immobilisation. But whether ZEN was actually immobilised in the soil in the present experiment cannot be clarified conclusively. The extraction method was valid to recover ZEN from soil samples (Mortensen et al. 2003; Muñoz et al. 2015), but this finding needs to be investigated further. Despite the fact, that high adsorption to the soil matrix is related to a reduced mobility of a substance in general (Goldberg and Angle 1985; Vereecken 2005), there is still evidence that strong adsorbing substances may be transported to drainage water under appropriate environmental conditions (Elmholt 2008). This aspect could not be confirmed within the present experiment as ZEN was not detected in the percolate. Consequently, our fourth hypothesis that both, DON and ZEN, respectively, leach from contaminated maize material into the soil and percolate can be fully confirmed only for DON. Leaching of ZEN from infected maize material was detected as well, but ZEN was detected neither in the soil nor in the percolate.

The impact of the soil fauna in the biological degradation of *Fusarium* toxins from contaminated maize stubbles turned out to be difficult to assess, which reflects the complexity of environmental soil systems. In this context, special regard was given to the influences of the temperature, which is a driving factor for the mechanisms of interaction between different functional and trophic levels within the soil food web. A temperature-dependent decline of DON and ZEN in residual maize material was observed in all treatments, showing higher reduction rates when microcosms were incubated in the long-term at 25 °C than in the short-term at 17 °C. A faunal interactive impact on the reduction of DON in maize material was observed after long-term incubation at elevated temperature of 25 °C. It is tentatively concluded from these results, that rising temperatures due to global warming could accelerate the biodegradation of *Fusarium* toxins leading to lower concentrations in residual plant material. This eventually might reduce the leaching of mycotoxins into the soil ecosystem and minimise a potential contamination risk to the environment. However, this has to be confirmed in further studies, in which also masked mycotoxins have to be included. Future changes in frequency and heaviness of rainfall events predicted from global warming might increase a run-off of DON from contaminated plant residues into the aquatic environment, which, in contrast to ZEN, occurred in the percolate after heavy short-term watering of the microcosms. Whether such a run-off could detrimentally affect the sustainability of the aquatic system appears questionable, as large dilution effects can be reasonably assumed.

## Supplementary information

Below is the link to the electronic supplementary material.

Supplementary file1 (DOCX 39 KB)
