# Supplementary material for: Effects of temperature and soil fauna on the reduction and leaching of deoxynivalenol and zearalenone from Fusarium graminearum-infected maize stubbles
Source: Mycotoxin Res. 2021 Jun 25;37(3):249–63. doi: 10.1007/s12550-021-00434-y (PMC8318931; doi:10.1007/s12550-021-00434-y)
Supplement: Supplementary file 1 — Supplementary file1 (DOCX 39 KB) [file 12550_2021_434_MOESM1_ESM.docx]

**Article title**

Effects of temperature and soil fauna on the reduction and leaching of deoxynivalenol (DON) and zearalenone (ZEN) in from *Fusarium graminearum*-infected maize stubbles

**Journal name**

Mycotoxin Research

**Author names**

Friederike Meyer-Wolfarth (corresponding author), Elisabeth Oldenburg, Torsten Meiners, Katherine Muñoz, Stefan Schrader

**Affiliation of the corresponding author**

Julius Kühn Institute (JKI) - Federal Research Centre for Cultivated Plants, Institute for Plant Protection in Field Crops and Grassland, Braunschweig, Germany

**E-mail address of the corresponding author**

[friederike.meyer-wolfarth@julius-kuehn.de](mailto:friederike.meyer-wolfarth@julius-kuehn.de)

**Supplementary material: Tables S1-S5**

**Table S1:** Original data of the initial concentrations of deoxynivalenol (DON) and zearalenone (ZEN) (µg kg^-1^) in contaminated maize stubbles before the beginning of the experiment.

| **Sample #** | **DON** | **ZEN** |
| --- | --- | --- |
| A1 | 10610.00 | 3130.00 |
| A2 | 11492.50 | 1999.25 |
| A3 | 10277.50 | 2867.50 |
| A4 | 9837.50 | 3005.00 |
| A5 | 10090.00 | 2899.00 |

**Table S2:** Original data of the concentrations of deoxynivalenol (DON) and zearalenone (ZEN) (µg kg^-1^) in contaminated maize stubbles on the soil surface of the microcosms before (Maize) and after (Maize leached) the leaching step in different faunal treatments: “Collembolans”, “Earthworm”, “Mix”, and non-faunal “Control” after 3 at the different temperatures (17°C and 25°C).

|  |  |  | **3 Weeks** | | | | | | |
| --- | --- | --- | --- | --- | --- | --- | --- | --- | --- |
|  |  |  | **17°C** | | | | | | |
| **Sample #** | **Treatment** |  | **Maize** | | |  | **Maize leached** | | |
|  |  |  | **DON** |  | **ZEN** |  | **DON** |  | **ZEN** |
| 41 | Collembolan |  | 4987.50 |  | 3302.50 |  | 1507.50 |  | 898.75 |
| 42 | Collembolan |  | 4040.00 |  | 2965.00 |  | 1160.00 |  | 194.50 |
| 43 | Collembolan |  | 3560.00 |  | 3372.50 |  | 428.00 |  | 1560.00 |
| 44 | Collembolan |  | 5140.00 |  | 2757.50 |  | 910.13 |  | 1484.50 |
| 45 | Collembolan |  | 4702.50 |  | 2742.50 |  | 517.50 |  | 1569.13 |
|  |  |  |  |  |  |  |  |  |  |
| 46 | Earthworm |  | 3917.50 |  | 3285.00 |  | 503.25 |  | 1411.75 |
| 47 | Earthworm |  | 3877.50 |  | 3387.50 |  | 416.00 |  | 1676.50 |
| 48 | Earthworm |  | 2470.00 |  | 2520.00 |  | 1365.00 |  | 1902.50 |
| 49 | Earthworm |  | 3465.00 |  | 2555.00 |  | 980.00 |  | 1957.50 |
| 50 | Earthworm |  | 4682.50 |  | 2840.00 |  | 541.25 |  | 1603.88 |
|  |  |  |  |  |  |  |  |  |  |
| 51 | Mix |  | 3385.00 |  | 2640.00 |  | 500.38 |  | 1649.13 |
| 52 | Mix |  | 3397.50 |  | 3342.50 |  | 517.50 |  | 1524.88 |
| 53 | Mix |  | 2707.50 |  | 3002.50 |  | 268.88 |  | 1378.88 |
| 54 | Mix |  | 2910.00 |  | 2705.00 |  | 1067.50 |  | 2480.00 |
| 55 | Mix |  | 3075.25 |  | 3301.00 |  | 1052.50 |  | 2399.50 |
|  |  |  |  |  |  |  |  |  |  |
| 56 | Control |  | 4240.00 |  | 2185.00 |  | 885.00 |  | 1550.25 |
| 57 | Control |  | 3027.50 |  | 3192.50 |  | 467.50 |  | 952.50 |
| 58 | Control |  | 2885.00 |  | 3630.00 |  | 641.25 |  | 1606.63 |
| 59 | Control |  | 3072.50 |  | 3440.00 |  | 805.50 |  | 1864.00 |
| 60 | Control |  | 4137.50 |  | 2767.50 |  | 601.25 |  | 1702.75 |
|  |  |  | **25°C** | | | | | | |
| 61 | Collembolan |  | 3124.50 |  | 2440.00 |  | 1257.50 |  | 1730.00 |
| 62 | Collembolan |  | 2970.00 |  | 2437.50 |  | 517.50 |  | 1695.00 |
| 63 | Collembolan |  | 2132.50 |  | 22745* |  | 440.38 |  | 1205.00 |
| 64 | Collembolan |  | 3485.00 |  | 1090.00 |  | 554.88 |  | 1071.00 |
| 65 | Collembolan |  | 2357.50 |  | 1815.00 |  | 355.63 |  | 1112.13 |
|  |  |  |  |  |  |  |  |  |  |
| 66 | Earthworm |  | 3557.50 |  | 2097.50 |  | 501.25 |  | 1344.00 |
| 67 | Earthworm |  | 3510.00 |  | 1615.00 |  | 472.00 |  | 1136.63 |
| 68 | Earthworm |  | 5112.50 |  | 2098.75 |  | 1019.50 |  | 1263.38 |
| 69 | Earthworm |  | 4272.50 |  | 1982.50 |  | < LOD |  | 842.50 |
| 70 | Earthworm |  | 1710.00 |  | 497.50 |  | 745.00 |  | 1187.50 |
|  |  |  |  |  |  |  |  |  |  |
| 71 | Mix |  | 3607.50 |  | 1976.00 |  | 363.00 |  | 1275.00 |
| 72 | Mix |  | 3765.00 |  | 2282.50 |  | 683.88 |  | 1539.25 |
| 73 | Mix |  | 3125.25 |  | 2645.00 |  | 621.25 |  | 1720.13 |
| 74 | Mix |  | 3065.00 |  | 2280.00 |  | 451.00 |  | 1597.50 |
| 75 | Mix |  | 3750.25 |  | 2657.50 |  | 520.00 |  | 900.25 |
|  |  |  |  |  |  |  |  |  |  |
| 76 | Control |  | 3567.50 |  | 2427.50 |  | 510.50 |  | 1382.88 |
| 77 | Control |  | 4112.50 |  | 1165.00 |  | 585.00 |  | 1732.50 |
| 78 | Control |  | 3495.00 |  | 1420.00 |  | 464.75 |  | 1503.38 |
| 79 | Control |  | 3362.50 |  | 2387.50 |  | 835.00 |  | 1847.50 |
| 80 | Control |  | 3317.50 |  | 328.25 |  | 715.00 |  | 1631000* |

LOD = limit of detection

*Data not included in the statistical analysis

**Table S3:** Original data of the concentrations of deoxynivalenol (DON) and zearalenone (ZEN) (µg kg^-1^) in contaminated maize stubbles on the soil surface of the microcosms before (maize) and after (maize leached) the leaching step in different faunal treatments: “Collembolans”, “Earthworm”, “Mix”, and non-faunal “Control” after 6 at the different temperatures (17°C and 25°C).

|  |  |  | **6 Weeks** | | | | | | |
| --- | --- | --- | --- | --- | --- | --- | --- | --- | --- |
|  |  |  | **17°C** | | | | | | |
| **Sample #** | **Treatment** |  | **Maize** | | |  | **Maize leached** | | |
|  |  |  | **DON** |  | **ZEN** |  | **DON** |  | **ZEN** |
| 1 | Collembolan |  | 2097.50 |  | 1290.00 |  | 405.00 |  | 885.00 |
| 2 | Collembolan |  | 1232.50 |  | 2477.50 |  | 552.88 |  | 720.25 |
| 3 | Collembolan |  | 750.00 |  | 623.50 |  | 223.13 |  | 742.63 |
| 4 | Collembolan |  | 2137.50 |  | 2715.00 |  | 174.88 |  | 1196.25 |
| 5 | Collembolan |  | 912.50 |  | 3207.50 |  | ND |  | ND |
|  |  |  |  |  |  |  |  |  |  |
| 6 | Earthworm |  | 4490.00 |  | 1192.50 |  | 461.75 |  | 812.25 |
| 7 | Earthworm |  | 1985.00 |  | 1672.50 |  | 627.38 |  | 741.75 |
| 8 | Earthworm |  | 2998.75 |  | 1335.00 |  | 551.13 |  | 514.50 |
| 9 | Earthworm |  | 4440.00 |  | 1202.50 |  | 1787.50 |  | 449.50 |
| 10 | Earthworm |  | 5792.50 |  | 2249.00 |  | 895.00 |  | 1557.50 |
|  |  |  |  |  |  |  |  |  |  |
| 11 | Mix |  | 3845.00 |  | 1470.00 |  | 642.88 |  | 646.88 |
| 12 | Mix |  | 2805.00 |  | 915.00 |  | 323.88 |  | 1019.75 |
| 13 | Mix |  | 4145.00 |  | 852.50 |  | 1065.63 |  | 838.63 |
| 14 | Mix |  | 3132.50 |  | 2001.00 |  | 1035.00 |  | 642.50 |
| 15 | Mix |  | 1795.00 |  | 1642.50 |  | 1255.00 |  | 349.75 |
|  |  |  |  |  |  |  |  |  |  |
| 16 | Control |  | 2085.00 |  | 1560.00 |  | 940.00 |  | 1565.00 |
| 17 | Control |  | 2592.50 |  | 1967.50 |  | 1687.50 |  | 580.00 |
| 18 | Control |  | 1792.50 |  | 2227.50 |  | 364.00 |  | 1096.13 |
| 19 | Control |  | 3422.50 |  | 2262.50 |  | 361.13 |  | 924.00 |
| 20 | Control |  | 1532.50 |  | 692.50 |  | 871.00 |  | 539.75 |
|  |  |  | **25°C** | | | | | | |
| 21 | Collembolan |  | 3587.50 |  | 150.75 |  | 1968.88 |  | 389.75 |
| 22 | Collembolan |  | 2124.00 |  | 1237.50 |  | 139.63 |  | 792.13 |
| 23 | Collembolan |  | 2035.00 |  | 792.50 |  | 477.50 |  | 801.25 |
| 24 | Collembolan |  | 3775.25 |  | 1310.00 |  | 2047.50 |  | 1180.00 |
| 25 | Collembolan |  | 1705.00 |  | 1152.50 |  | 286.50 |  | 415.00 |
|  |  |  |  |  |  |  |  |  |  |
| 26 | Earthworm |  | 415.00 |  | 1147.50 |  | 135.63 |  | 1038.75 |
| 27 | Earthworm |  | 2950.00 |  | 1552.50 |  | 414.88 |  | 1153.13 |
| 28 | Earthworm |  | 1175.75 |  | 1272.50 |  | 306.25 |  | 506.75 |
| 29 | Earthworm |  | 5352.00 |  | 1032.50 |  | 830.00 |  | 777.50 |
| 30 | Earthworm |  | 1302.50 |  | 139.75 |  | 1212.50 |  | 877.50 |
|  |  |  |  |  |  |  |  |  |  |
| 31 | Mix |  | ND |  | ND |  | 652.50 |  | 136.25 |
| 32 | Mix |  | 194.00 |  | 337.50 |  | < LOD |  | 460.00 |
| 33 | Mix |  | 942.50 |  | 887.50 |  | 286.88 |  | 522.25 |
| 34 | Mix |  | 1060.00 |  | 1047.50 |  | < LOD |  | 291.88 |
| 35 | Mix |  | 355.75 |  | 902.50 |  | 128.50 |  | 749.63 |
|  |  |  |  |  |  |  |  |  |  |
| 36 | Control |  | 5045.00 |  | 1062.50 |  | ND |  | ND |
| 37 | Control |  | 3512.50 |  | 1052.50 |  | ND |  | ND |
| 38 | Control |  | 4025.25 |  | 206.25 |  | 1943.63 |  | 206.75 |
| 39 | Control |  | 1687.50 |  | 667.50 |  | 1279.25 |  | 598.00 |
| 40 | Control |  | 1702.50 |  | 1142.50 |  | 80.00 |  | 898.75 |

LOD = limit of detection

ND = No data

**Table S4:** Original data of the concentrations of deoxynivalenol (DON) and zearalenone (ZEN) in percolate (eluted water) (µg L^-1^) and soil (µg kg^-1^) of the microcosms before (soil) and after (soil leached) the leaching step in different faunal treatments: “Collembolans”, “Earthworm”, “Mix”, and non-faunal “Control” after 3 at the different temperatures (17°C and 25°C).

|  |  |  | **3 Weeks** | | | | | | | | | | |
| --- | --- | --- | --- | --- | --- | --- | --- | --- | --- | --- | --- | --- | --- |
|  |  |  | **17°C** | | | | | | | | | | |
| **Sample #** | **Treatment** |  | **Percolate** | | |  | **Soil** | | |  | **Soil leached** | | |
|  |  |  | **DON** |  | **ZEN** |  | **DON** |  | **ZEN** |  | **DON** |  | **ZEN** |
| 41 | Collembolan | | 149.78 |  | < LOD |  | < LOD |  | < LOD |  | < LOD |  | < LOD |
| 42 | Collembolan | | 147.4 |  | < LOD |  | < LOD |  | < LOD |  | 1.77 |  | < LOD |
| 43 | Collembolan | | 132.78 |  | < LOD |  | < LOD |  | < LOD |  | 1.34 |  | < LOD |
| 44 | Collembolan | | 174.33 |  | < LOD |  | < LOD |  | < LOD |  | 3.81 |  | < LOD |
| 45 | Collembolan | | 171.45 |  | < LOD |  | < LOD |  | < LOD |  | < LOD |  | < LOD |
|  |  |  |  |  |  |  |  |  |  |  |  |  |  |
| 46 | Earthworm |  | 188.48 |  | < LOD |  | < LOD |  | < LOD |  | 0.92 |  | < LOD |
| 47 | Earthworm |  | 261.09 |  | < LOD |  | < LOD |  | < LOD |  | 2.13 |  | < LOD |
| 48 | Earthworm |  | 256.79 |  | < LOD |  | 2.39 |  | < LOD |  | < LOD |  | 1.62 |
| 49 | Earthworm |  | 239.44 |  | < LOD |  | < LOD |  | < LOD |  | 3.15 |  | < LOD |
| 50 | Earthworm |  | 295.57 |  | < LOD |  | < LOD |  | < LOD |  | 2.27 |  | < LOD |
|  |  |  |  |  |  |  |  |  |  |  |  |  |  |
| 51 | Mix |  | 253.9 |  | < LOD |  | < LOD |  | < LOD |  | < LOD |  | 1.66 |
| 52 | Mix |  | 230.08 |  | < LOD |  | < LOD |  | < LOD |  | < LOD |  | < LOD |
| 53 | Mix |  | 177.58 |  | < LOD |  | < LOD |  | < LOD |  | < LOD |  | < LOD |
| 54 | Mix |  | 199.83 |  | < LOD |  | < LOD |  | < LOD |  | < LOD |  | < LOD |
| 55 | Mix |  | 178.41 |  | < LOD |  | < LOD |  | < LOD |  | 1.98 |  | < LOD |
|  |  |  |  |  |  |  |  |  |  |  |  |  |  |
| 56 | Control |  | 198.48 |  | < LOD |  | < LOD |  | < LOD |  | 1.9 |  | < LOD |
| 57 | Control |  | 116.82 |  | < LOD |  | < LOD |  | < LOD |  | < LOD |  | < LOD |
| 58 | Control |  | 139.33 |  | < LOD |  | < LOD |  | < LOD |  | 2.49 |  | < LOD |
| 59 | Control |  | 216.63 |  | < LOD |  | < LOD |  | < LOD |  | < LOD |  | < LOD |
| 60 | Control |  | 228.49 |  | < LOD |  | < LOD |  | < LOD |  | 2.79 |  | < LOD |
|  |  |  | **25°C** | | | | | | | | | | |
| 61 | Collembolan | | 118.86 |  | < LOD |  | < LOD |  | < LOD |  | 1.71 |  | 1.43 |
| 62 | Collembolan | | 78.53 |  | < LOD |  | < LOD |  | < LOD |  | 1.4 |  | < LOD |
| 63 | Collembolan | | 130.73 |  | < LOD |  | < LOD |  | < LOD |  | 0.63 |  | < LOD |
| 64 | Collembolan | | 111.48 |  | < LOD |  | < LOD |  | < LOD |  | 0.94 |  | < LOD |
| 65 | Collembolan | | 111.46 |  | < LOD |  | < LOD |  | < LOD |  | 2.79 |  | < LOD |
|  |  |  |  |  |  |  |  |  |  |  |  |  |  |
| 66 | Earthworm |  | 192.22 |  | < LOD |  | < LOD |  | < LOD |  | 2.15 |  | 0.86 |
| 67 | Earthworm |  | 165.62 |  | < LOD |  | < LOD |  | < LOD |  | < LOD |  | < LOD |
| 68 | Earthworm |  | 239.89 |  | < LOD |  | < LOD |  | < LOD |  | < LOD |  | < LOD |
| 69 | Earthworm |  | 202.56 |  | < LOD |  | < LOD |  | < LOD |  | < LOD |  | < LOD |
| 70 | Earthworm |  | 53.86 |  | < LOD |  | < LOD |  | < LOD |  | < LOD |  | 1.2 |
|  |  |  |  |  |  |  |  |  |  |  |  |  |  |
| 71 | Mix |  | 250.69 |  | < LOD |  | 9.28 |  | < LOD |  | 2.59 |  | < LOD |
| 72 | Mix |  | 217.48 |  | < LOD |  | 32.87 |  | < LOD |  | 4.23 |  | 1.75 |
| 73 | Mix |  | 190.12 |  | < LOD |  | < LOD |  | < LOD |  | 4.25 |  | 0.68 |
| 74 | Mix |  | 179.66 |  | < LOD |  | < LOD |  | < LOD |  | 2.17 |  | 0.76 |
| 75 | Mix |  | 240.17 |  | < LOD |  | < LOD |  | < LOD |  | 3.28 |  | < LOD |
|  |  |  |  |  |  |  |  |  |  |  |  |  |  |
| 76 | Control |  | 163.01 |  | < LOD |  | 33.1 |  | < LOD |  | 2.16 |  | < LOD |
| 77 | Control |  | 203.83 |  | < LOD |  | < LOD |  | < LOD |  | < LOD |  | < LOD |
| 78 | Control |  | 197.84 |  | < LOD |  | < LOD |  | < LOD |  | < LOD |  | < LOD |
| 79 | Control |  | 159.71 |  | < LOD |  | < LOD |  | < LOD |  | 4.55 |  | < LOD |
| 80 | Control |  | 154.27 |  | < LOD |  | < LOD |  | < LOD |  | 3.52 |  | < LOD |

LOD = limit of detection

ND = No data

**Table S5:** Original data of the concentrations of deoxynivalenol (DON) and zearalenone (ZEN) in percolate (eluted water) (µg L^-1^) and soil (µg kg^-1^) of the microcosms before (soil) and after (soil leached) the leaching step in different faunal treatments: “Collembolans”, “Earthworm”, “Mix”, and non-faunal “Control” after 6 at the different temperatures (17°C and 25°C).

|  |  |  | **6 Weeks** | | | | | | | | | | |
| --- | --- | --- | --- | --- | --- | --- | --- | --- | --- | --- | --- | --- | --- |
| **Sample #** | **Treatment** |  | **17°C** | | | | | | | | | | |
|  |  |  | **Percolate** | | |  | **Soil** | | |  | **Soil leached** | | |
|  |  |  | **DON** |  | **ZEN** |  | **DON** |  | **ZEN** |  | **DON** |  | **ZEN** |
| 1 | Collembolan | | ND |  | < LOD |  | 11.14 |  | < LOD |  | 22.61 |  | < LOD |
| 2 | Collembolan | | 47.53 |  | < LOD |  | 11.48 |  | < LOD |  | 9.85 |  | < LOD |
| 3 | Collembolan | | 42.76 |  | < LOD |  | 9.25 |  | < LOD |  | 15.13 |  | < LOD |
| 4 | Collembolan | | 17.86 |  | < LOD |  | 10.34 |  | < LOD |  | 20.46 |  | < LOD |
| 5 | Collembolan | | 73.48 |  | < LOD |  | 7.48 |  | < LOD |  | bld |  | < LOD |
|  |  |  |  |  |  |  |  |  |  |  |  |  |  |
| 6 | Earthworm |  | 112.66 |  | < LOD |  | 14.05 |  | < LOD |  | 6.03 |  | < LOD |
| 7 | Earthworm |  | 25.65 |  | < LOD |  | 8.92 |  | < LOD |  | 14.44 |  | < LOD |
| 8 | Earthworm |  | 101.09 |  | < LOD |  | 10.84 |  | < LOD |  | 9.88 |  | < LOD |
| 9 | Earthworm |  | 21.34 |  | < LOD |  | 11.00 |  | < LOD |  | 21.49 |  | < LOD |
| 10 | Earthworm |  | 165.41 |  | < LOD |  | 9.13 |  | < LOD |  | 6.27 |  | < LOD |
|  |  |  |  |  |  |  |  |  |  |  |  |  |  |
| 11 | Mix |  | 107.70 |  | < LOD |  | 10.03 |  | < LOD |  | bld |  | < LOD |
| 12 | Mix |  | 178.39 |  | < LOD |  | 4.03 |  | < LOD |  | 15.24 |  | < LOD |
| 13 | Mix |  | ND |  | < LOD |  | < LOD |  | < LOD |  | < LOD |  | < LOD |
| 14 | Mix |  | 55.14 |  | < LOD |  | 13.08 |  | < LOD |  | 16.63 |  | < LOD |
| 15 | Mix |  | 37.24 |  | < LOD |  | ND |  | ND |  | 9.55 |  | < LOD |
|  |  |  |  |  |  |  |  |  |  |  |  |  |  |
| 16 | Control |  | 88.28 |  | < LOD |  | 9.20 |  | < LOD |  | 10.30 |  | < LOD |
| 17 | Control |  | 53.33 |  | < LOD |  | < LOD |  | < LOD |  | 8.43 |  | < LOD |
| 18 | Control |  | 74.86 |  | < LOD |  | 7.71 |  | < LOD |  | < LOD |  | < LOD |
| 19 | Control |  | 102.70 |  | < LOD |  | 5.89 |  | < LOD |  | < LOD |  | < LOD |
| 20 | Control |  | 78.27 |  | < LOD |  | 7.16 |  | < LOD |  | < LOD |  | < LOD |
|  |  |  | **25°C** | | | | | | | | | | |
| 21 | Collembolan | | 77.69 |  | < LOD |  | 12.25 |  | < LOD |  | 4.77 |  | < LOD |
| 22 | Collembolan | | 40.23 |  | < LOD |  | 5.62 |  | < LOD |  | < LOD |  | < LOD |
| 23 | Collembolan | | 58.10 |  | < LOD |  | 9.27 |  | < LOD |  | 16.30 |  | < LOD |
| 24 | Collembolan | | 102.66 |  | < LOD |  | 11.47 |  | < LOD |  | < LOD |  | < LOD |
| 25 | Collembolan | | 65.20 |  | < LOD |  | 10.86 |  | < LOD |  | < LOD |  | < LOD |
|  |  |  |  |  |  |  |  |  |  |  |  |  |  |
| 26 | Earthworm |  | 57.21 |  | < LOD |  | 10.28 |  | < LOD |  | < LOD |  | < LOD |
| 27 | Earthworm |  | 32.63 |  | < LOD |  | < LOD |  | < LOD |  | 5.58 |  | < LOD |
| 28 | Earthworm |  | 55.07 |  | < LOD |  | 10.13 |  | < LOD |  | < LOD |  | < LOD |
| 29 | Earthworm |  | 87.40 |  | < LOD |  | 6.97 |  | < LOD |  | < LOD |  | < LOD |
| 30 | Earthworm |  | 76.13 |  | < LOD |  | 9.27 |  | < LOD |  | < LOD |  | < LOD |
|  |  |  |  |  |  |  |  |  |  |  |  |  |  |
| 31 | Mix |  | ND |  | ND |  | 7.58 |  | < LOD |  | 6.72 |  | < LOD |
| 32 | Mix |  | ND |  | ND |  | 10.55 |  | < LOD |  | 12.09 |  | < LOD |
| 33 | Mix |  | 24.21 |  | < LOD |  | 12.41 |  | < LOD |  | < LOD |  | < LOD |
| 34 | Mix |  | 1.02 |  | < LOD |  | ND |  | ND |  | 16.91 |  | < LOD |
| 35 | Mix |  | 12.14 |  | < LOD |  | < LOD |  | < LOD |  | 9.22 |  | < LOD |
|  |  |  |  |  |  |  |  |  |  |  |  |  |  |
| 36 | Control |  | ND |  | ND |  | 8.82 |  | < LOD |  | 7.90 |  | < LOD |
| 37 | Control |  | ND |  | ND |  | 10.34 |  | < LOD |  | 12.64 |  | < LOD |
| 38 | Control |  | 30.20 |  | < LOD |  | 11.43 |  | < LOD |  | 11.06 |  | < LOD |
| 39 | Control |  | 23.78 |  | < LOD |  | 10.10 |  | < LOD |  | < LOD |  | < LOD |
| 40 | Control |  | 29.66 |  | < LOD |  | ND |  | ND |  | 5.26 |  | < LOD |

LOD = limit of detection

ND = No data
